# Impact of dose reporting modes on dosimetric accuracy in a national end-to-end stereotactic spine radiotherapy audit^☆^

**DOI:** 10.1016/j.phro.2026.101041

**Published:** 2026-07-14

**Authors:** Rushil Patel, Mohammad Hussein, Jonny Lee, Patricia Diez, Ileana Silvestre Patallo, Hannah Cook, Alexandros Douralis, Catharine H. Clark

**Affiliations:** a National Radiotherapy Trials Quality Assurance Group, Mount Vernon Cancer Centre, Radiotherapy Physics Northwood, United Kingdom; b National Physical Laboratory, Metrology for Medical Physics Teddington, United Kingdom; c Dorset Cancer Centre, Radiotherapy, Poole, United Kingdom; d University College London Hospitals NHS Foundation Trust, London, United Kingdom; e University College London, London, UK

**Keywords:** SABR, SBRT, Audit, Dosimetry, Alanine, Radiochromic film, Dose to Medium

## Abstract

**Background and purpose:**

Spinal Stereotactic Body Radiotherapy (SBRT) is among the most complex treatments currently delivered. A multi-centre dosimetry audit evaluated the accuracy of spinal SBRT treatment delivery and the impact of different dose reporting modes (DRM).

**Methods and materials:**

The audit used anthropomorphic phantoms containing alanine in the Planning Target Volume (PTV) and Gafchromic™ EBT3 film in the axial plane, with a combination of onsite and remote audits due to Covid travel restrictions. Centres used their local planning protocol and technique to create a clinically acceptable treatment plan, following national guidance.

**Results:**

Fifty-nine plans from 44 treatment planning systems at 41 centres were analysed. The mean PTV dose difference was 1.2% [−6.5–8.5%] with alanine and 1.0% [−7.0–8.5%] within the 100% isodose for film. Alanine measurements were, on average, higher than the calculated dose for all plans, but lower for the D_w,m_ specifically. For alanine and film, the choice of DRM was statistically significant.

The mean distance to agreement and average Centre of Dose difference for films was 0.82 mm [0.35–1.7 mm] and 0.78 mm [0.1–1.7 mm] respectively. The choice of DRM was not significant for either metric.

**Conclusions:**

This audit demonstrated that the majority of centres are delivering spinal SBRT consistently, with acceptable dosimetric and geometric accuracy. However, it also highlighted the challenges of verifying doses reported in D_m,m_ and D_w,m_, as there was a statistically significant difference with the measured dose when stratified by DRM.

## Highlights

• Spinal radiotherapy showed high spatial accuracy with a mean offset of 0.78 mm.
• Across 59 audited plans, the mean alanine target dose difference was 1.2%.
• Dose reporting modes caused significant mean film dose variations of up to 2.6%.
• Film dose measurements mirrored alanine outcomes, yielding a 1.0% mean difference.

## Graphical abstract

**Figure:**
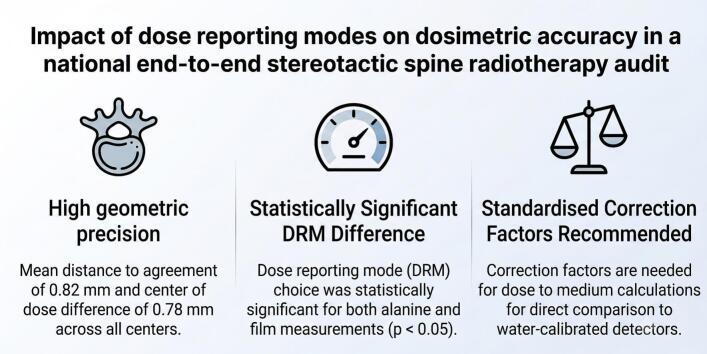

## 1 Introduction

Stereotactic Body Radiotherapy (SBRT) uses higher doses in fewer fractions than conventional treatments to achieve effective cancer outcomes [1]. Spinal SBRT is particularly complex as the proximity of the spinal cord to the planning target volume (PTV) necessitates steep-dose gradients [2], [3], [4]. These sharp gradients pose a challenge for accurate dose delivery and measurement [5], [6], exacerbated by the complex geometries and composition of spinal PTVs.

SBRT guidelines [7], [8] recommend independent quality assurance, including dosimetry audits. For centres treating technically simpler SBRT sites (lung, bone and node), a lung SBRT audit had been performed [9]. For more complex SBRT indications (spine, liver, adrenal and pancreas), a spine SBRT audit was developed to provide an independent end-to-end check of the imaging, planning, verification and delivery of treatment, to ensure acceptable geometric and dosimetric accuracy across the audited centres, for all commonly used treatment platforms (Robotic stereotactic radiosurgery system, C-arm and ring Linacs).

During the audit, some centres were transitioning from type B to type C dose calculation algorithms. While radiotherapy dosimetry equipment is calibrated in terms of dose-to-water in water (D_w,w_), treatment planning systems (TPS) using type C algorithms can report calculated dose as dose-to-medium in a medium (D_m,m_) or dose-to-water in a medium (D_w,m_). These dose reporting modes (DRMs) create additional challenges as centres must choose which to report in denser media such as bone, where the difference between the two may be greater [10].

This study aimed to measure SBRT plans across multiple platforms to determine if significant differences exist between DRMs, and whether correction factors or alternative tolerances are warranted for national spine SBRT audits.

## 2 Methods and materials

### 2.1 Scanning and planning the phantom

The dosimetric audit was designed as a full end-to-end check of SBRT delivery using alanine and Gafchromic™ EBT3 film (Ashland, Wilmington, DE) in a customised CIRS E2E SBRT Thorax phantom (Sun Nuclear, Melbourne, FL). Initially offered as an onsite audit to 17 centres, it was converted to a postal audit for the additional 27 centres due to Covid pandemic restrictions. Fifty-nine plans from 41 centres were audited utilising 44 TPSs: 21 Eclipse (Varian, USA), 6 Pinnacle (Philips, Netherlands), 5 Precision (Accuray, USA), 8 RayStation (RaySearch, Sweden), and 4 Monaco (Elekta, Sweden) TPSs. DRMs comprised 38 D_w,w_, 9 D_w,m_ and 12 D_m,m_. As the original phantom was not considered suitable for shipping, a second, customised anthropomorphic phantom (ATOM phantom, CIRS, USA) was used for the postal audit. Both proprietary, tissue-equivalent epoxy phantoms featured custom recesses accommodating a cylindrical alanine holder within the PTV, film through the axial plane of the PTV, and had alignment pins inserted. The approach for both audits followed a similar process.

**Fig. 1:**
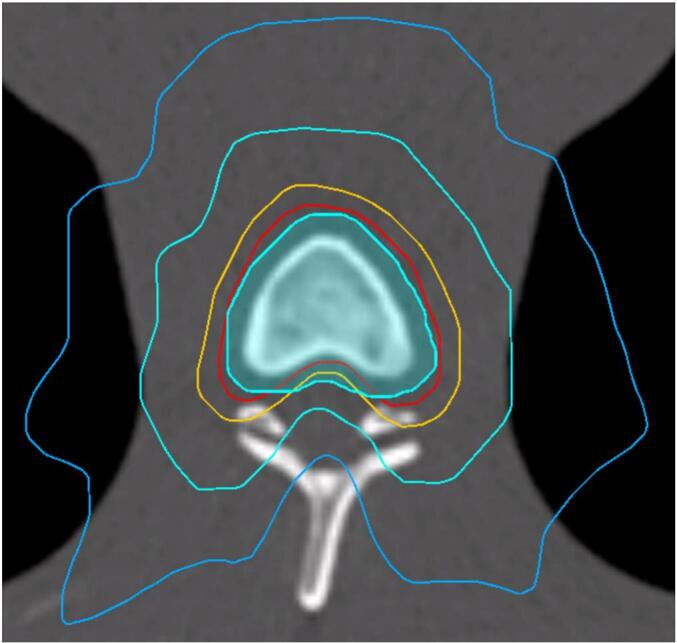
An example audit plan on the axial plane in the CIRS e2e SBRT phantom. PTV: Cyan, Isodose lines: 100% (red), 80% (yellow) 50% (light blue), 30% (dark blue). (For interpretation of the references to colour in this figure legend, the reader is referred to the web version of this article.)

A computed tomography (CT) scan of the relevant phantom was sent in advance to the centres to allow creation of a plan following national guidance [8]. Plan goals were: 27 Gy in three fractions, PTV 100% ≥ 95%, and normalised for optimal coverage whilst meeting mandatory dose constraints [11], using a dose grid ≤2 mm. Clinical target volumes (CTV) were 8.2 cm^3^ (onsite) and 24.4 cm^3^ (remote), with centres applying their clinical PTV expansion. An example plan is shown in Fig. 1. Centres CT-scanned the phantom on the day of the audit, transferred the prepared plan onto the new image and recalculated using all available dose calculation algorithms and DRMs. Onsite, auditors loaded the phantom with alanine and film prior to irradiation, while detectors were preloaded for the remote audit.

Machine reference output measurements were acquired before phantom setup on the treatment unit. Centres used their local image-matching protocol before delivering one fraction of the treatment. Measurements were acquired using alanine in the vertebral PTV adjacent to Gafchromic™ film in the axial plane of the treated vertebra. Radiotherapy data was collected for comparison with the measured data. For Equipment, algorithms and DRM combinations see Supplementary Table S1.

### 2.2 Reference and phantom measurements

For onsite audits, output measurements were acquired in local reference conditions using water-equivalent blocks. A 0.125cm^3^ Type-31,010 semiflex-ionisation-chamber (PTW, Germany) was used for the onsite audits, calibrated against the national primary standard at the National Physical Laboratory (NPL), Teddington, UK. For the remote audit, similarly calibrated alanine discs in a Farmer-shaped holder provided by NPL were used. Four 5 cm × 5 cm squares of Gafchromic™ film were irradiated in reference conditions at various doses for local film dose-calibration.

Both audits performed alanine dose measurements in a custom-designed holder made of the proprietary vertebral bone-tissue-equivalent epoxy used in the phantoms, holding 3 alanine pellets (each 2.3 mm thick and 5 mm in diameter). The holder was placed within the vertebral PTV in both phantoms, abutting the film. The alanine was traceable to the UK primary standard independently of the ion chamber chain. At 10 Gy, the uncertainty on the alanine measurements was 1.7% (k = 2) and pellet-to-pellet reproducibility was 1.0% (k = 2) [12].

Reference films were irradiated in the same conditions, on the same treatment unit used for the audit and therefore provided daily output normalisation. The main audit film was positioned axially between two slabs of the phantom through the PTV for both audits. Pins pierced the film and were visible on CT, facilitating the alignment of the measured dose with the calculated dose. Centres submitted the full DICOM RT dataset (CT scan, structures and dose files) to NPL for analysis. Profile evaluation and gamma analysis for the film measurements were undertaken using VIGO [13], an independent MATLAB film analysis software programme developed at NPL. For further details of VIGO methodology; see supplementary material a

### 2.3 Detector readout and analysis

Irradiated alanine pellets were read at NPL using an electron paramagnetic resonance spectrometer. Calibration factors for each alanine batch were determined at NPL and the results were calculated in terms of absorbed dose (Gy) and corrected for daily output using the mean dose from the reference output measurement. Each output-corrected alanine pellet dose was compared with the expected dose and mean differences were calculated.

Films were scanned five times using a 10000XL scanner (Epson, Japan) and averaged to minimise scanning variations. The average optical densities (OD) were calculated using the green channel for each calibration film to create a calibration curve of OD versus dose. Film analysis was performed by aligning measured and calculated dose using the pin marks in the film and the corresponding pin locations in the dose grid, with typical accuracy of ≤1 mm.

Gamma analysis was performed on the film area enclosed by 50% of the prescription isodose, this was selected to focus analysis on the high-dose-gradient regions and PTV. These are critical for evaluating PTV coverage and healthy tissue sparing, thus preventing inflation of gamma pass values from the low dose areas. The pass rate with gamma <1 was calculated using parameters of 5%/1 mm and 3%/2 mm global gamma, with dose normalised to the prescription dose. The centre of dose (COD) was calculated by taking the dose-weighted average position of each dose point inside the 100% isodose line, the difference between film and TPS COD was reported. Distance-to-agreement (DTA) was defined as <1% local dose difference between film and TPS within the 50% prescription isodose. Finally, the mean dose difference between film and TPS within the 100% prescription isodose was calculated.

Published recommendations for intensity modulated radiotherapy (IMRT) quality assurance (QA) [14] include use of gamma analysis with a recommended tolerance of 3%/2 mm. These values may be unsuitable for SBRT plans where there is a higher requirement for geometric precision at the expense of greater dose inhomogeneity. A combination of dose metrics may, therefore, be more useful to assess SBRT deliverability. A correlation matrix was generated using Pearson correlation coefficients for all of the audit metrics to determine which combination of metrics would be most useful for future reporting.

To visually compare the effects of the change in DRM/dose calculation algorithm on individual plans, plans were recalculated using all available algorithms/DRMs, mean dose difference from alanine and film against expected TPS dose were plotted.

## 3 Results

**Fig. 2:**
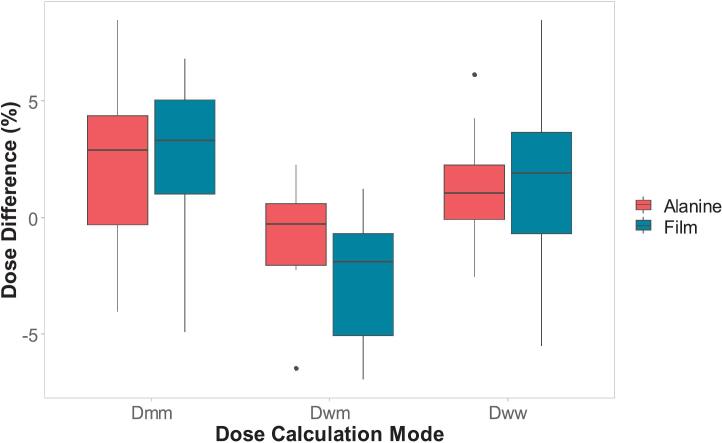
Alanine and film dose differences from TPS calculations in vertebral PTV within the 100% isodose, stratified by dose reporting mode (Dw,w/Dw,m/Dm,m). The boxes represent the interquartile range (IQR, 25th to 75th percentiles), and the central horizontal line indicates the median. Whiskers denote 1.5 times IQR, and individual points represent outlier data.

Mean dose difference in the PTV between alanine measured and TPS expected dose was 1.2% [−6.5% to 8.5%] for all algorithms, 92% of measurements were within ±5% of expected dose, with 76% of measurements within ±3%. When stratified by DRM (Fig. 2), mean dose differences were 2.5% (D_m,m_), −0.8% (D_w,m_) and 1.3% (D_w,w_). Only one centre recorded a dose lower than the expected with deviation >3% (D_m,m_ and D_w,m_). Choice of DRM was statistically significant (chi-squared = 6.79, *p* = 0.03).

Mean dose difference measured within the 100% isodose for film was 1.0% [−7.0% to 8.5%] for all algorithms, 81% and 54% of measurements were within ±5% and ± 3% of the expected dose respectively. When stratified by DRM (Fig. 2), the mean dose differences were 2.6% (D_m,m_), −2.5% (D_w,m_) and 1.3% (D_w,w_). Five centres recorded a film dose lower than calculated of >3%. The choice of DRM was statistically significant (chi-squared = 11.26, *p* < 0.01).

**Fig. 3:**
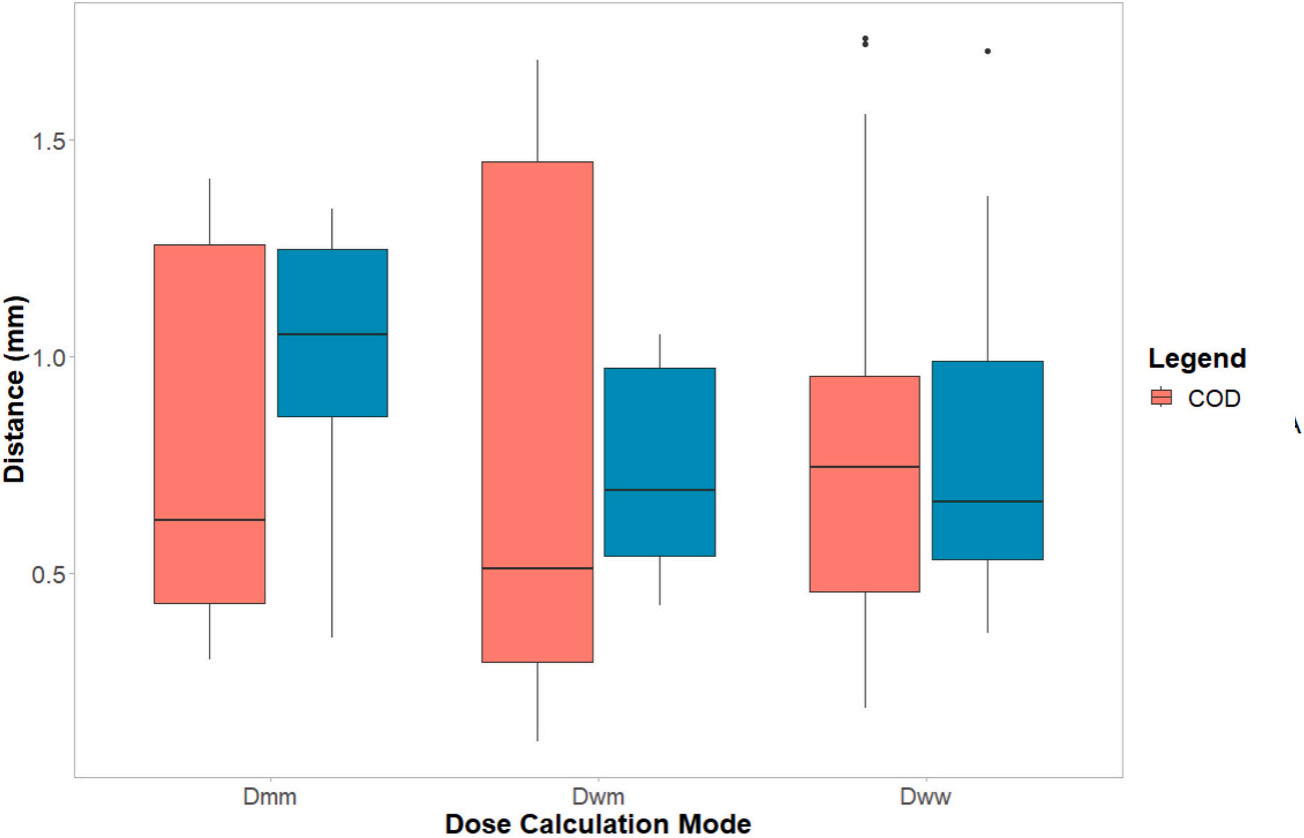
Mean DTA and Centre of Dose stratified by dose reporting mode (D_w,w_/D_w,m_/D_m,m_). The boxes represent the interquartile range (IQR, 25th to 75th percentiles), and the central horizontal line indicates the median. Whiskers denote 1.5 times IQR, and individual points represent outlier data.

Mean DTA from film measurements was 0.82 mm [0.35–1.7 mm]. When stratified by D_m,m,_ D_w,m_ and D_w,w_ these were 1.0 mm, 0.8 mm and 0.8 mm respectively (Fig. 3). Choice of DRM was not statistically significant (chi-squared = 4.82, *p* = 0.09). Seventy-one percent of centres achieved a treatment accuracy of <1 mm and all centres were within 2 mm. All audited centres used a PTV/ planning organ at risk volume (PRV) margin between 2 and 3 mm.

Average COD difference from film measurements was 0.78 mm [0.1–1.7 mm] with 73% of plans having a COD <1 mm. When stratified by D_m,m,_ D_w,m_ and D_w,w_ these were 0.8 mm, 0.8 mm and 0.8 mm, respectively (Fig. 3). Choice of DRM was not statistically significant for COD (chi-squared = 0.49, *p* = 0.78).

**Fig. 4:**
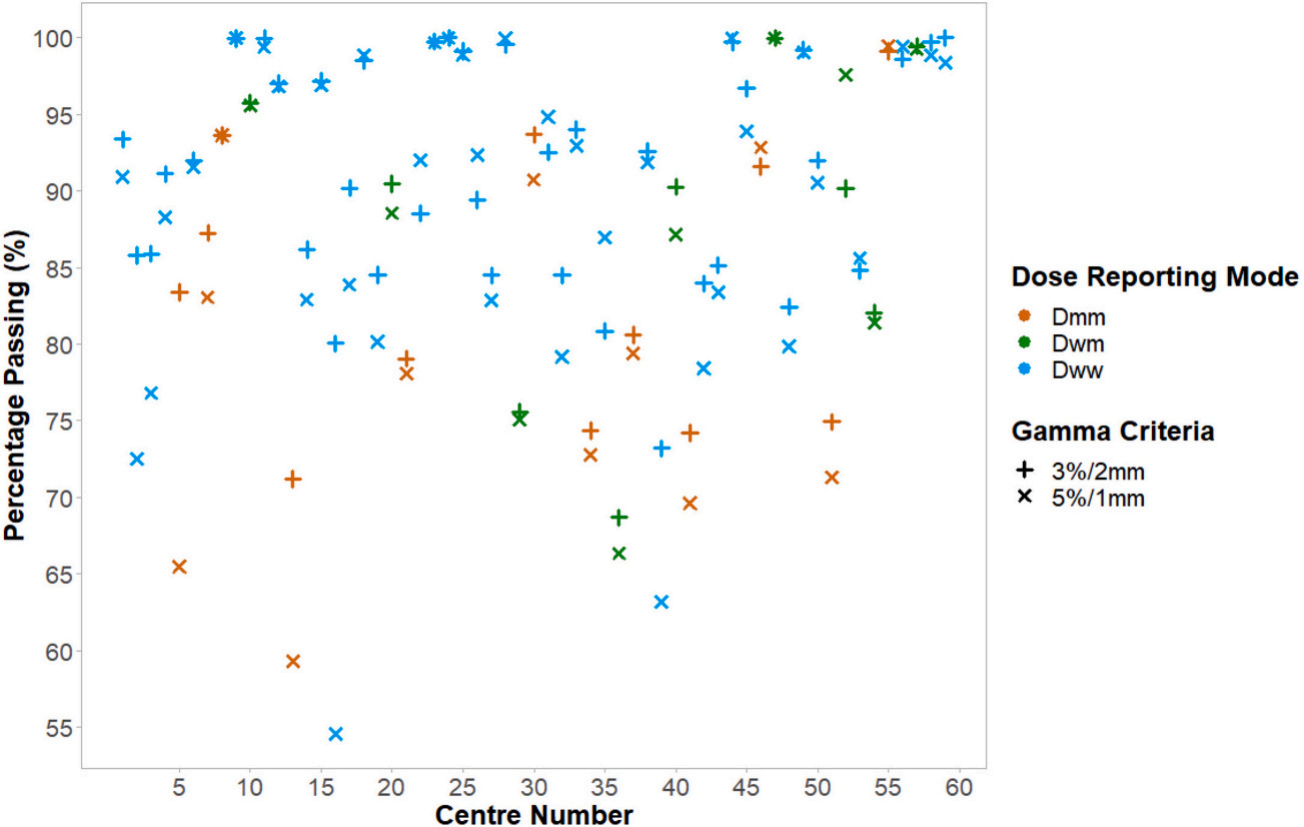
Global gamma analysis calculated at 3%/2 mm and 5%/1 mm for all centres colour mapped by dose reporting mode.

Gamma analysis of film vs TPS (Fig. 4) had a mean pass rate of 89.5% [68.7–100%] and 87.2% [54.6–100%] for 3%/2 mm and 5%/1 mm, respectively. When stratified by D_m,m,_ D_w,m_ and D_w,w_, these were 83.6%, 88.0% and 91.6% (3%/2 mm) and 79.6%, 87.9% and 89.4% (5%/1 mm) respectively. Choice of DRM was statistically significant for 3%/2 mm (chi-squared = 6.81, *p* = 0.03), but not for 5%/1 mm (chi-squared = 5.82, *p* = 0.06). For median and IQR values for all metrics; see Supplementary Table S2.

**Fig. 5:**
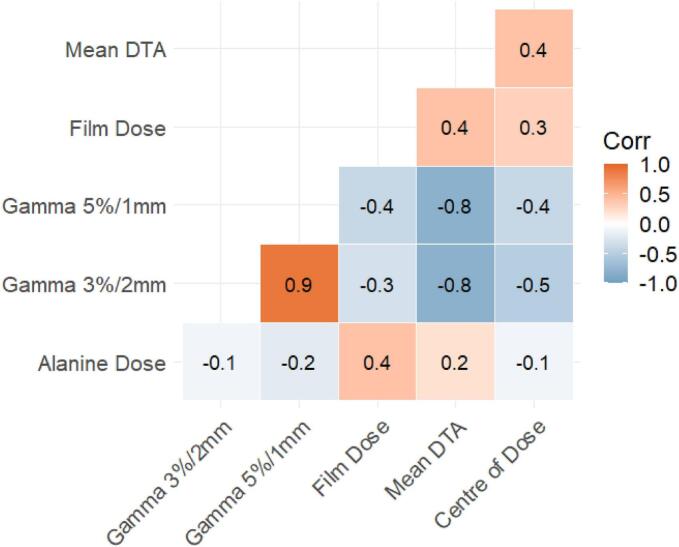
A correlation matrix visualizing the relationships between audit measurements, with colour intensity indicating the strength and direction of the Pearson correlation coefficient.

Pearson correlation coefficients between the evaluated dose metrics reached a maximum of r≈0.9 between the 3%/2 mm and 5%/1 mm gamma criteria, whilst alanine dose and COD yielded the lowest correlation coefficients (Fig. 5).

**Fig. 6:**
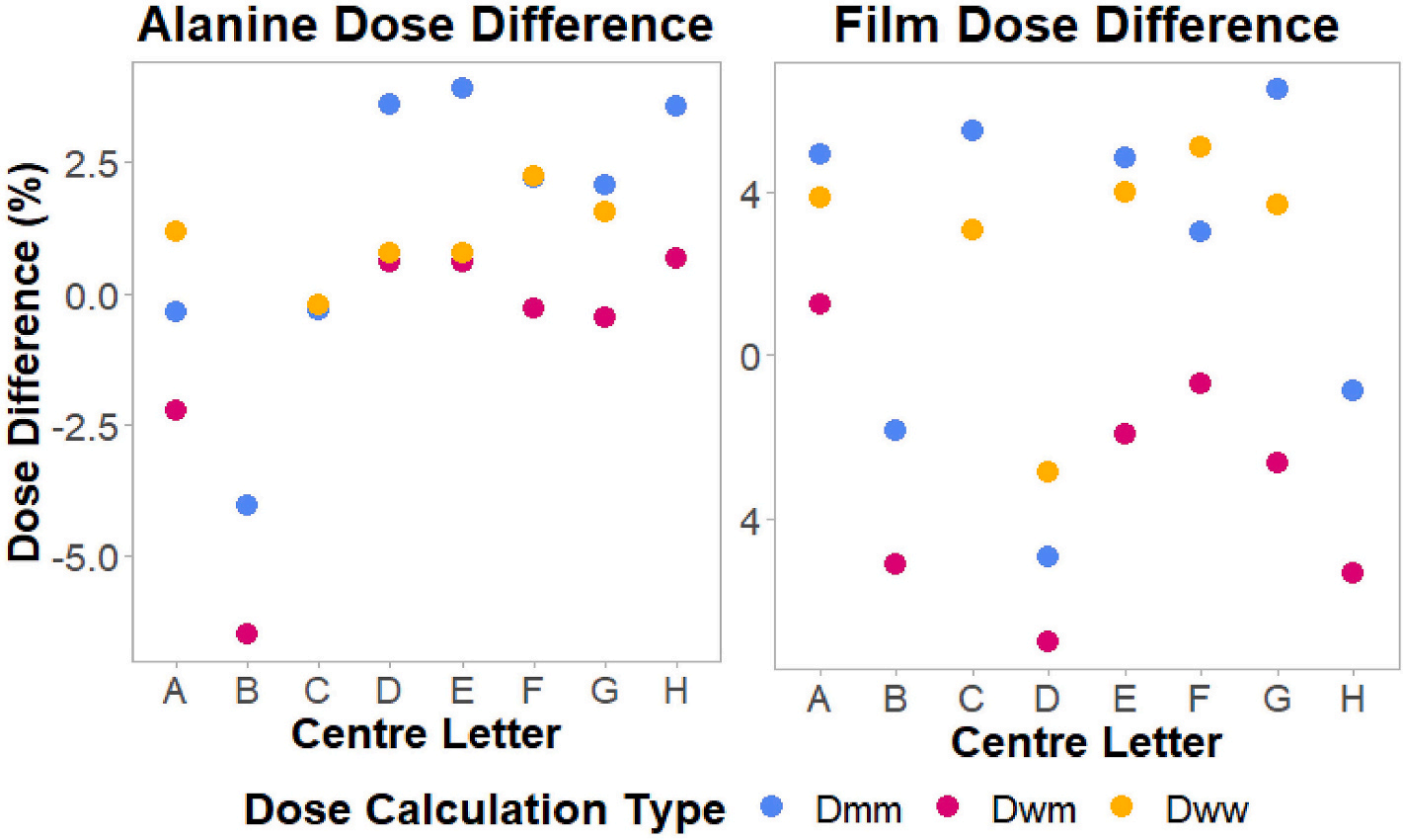
Alanine and mean film dose difference for single plans recalculated using all available DRMs and algorithms. Positive values indicate measuring hotter than planned, whilst negative indicate measuring colder than planned.

For the subset of individual plans recalculated using multiple dose reporting modes, the mean measured dose differences relative to expected values ranged from −2.5% for D_w,m_ up to 2.6% for D_m,m_ (Fig. 6).

## 4 Discussion

This multi-centre audit evaluated the delivery accuracy of spinal SBRT using anthropomorphic phantoms with alanine and gafchromic film across 59 plans. Overall geometric agreement was high, with mean differences in DTA and COD remaining below 1 mm, indicating high geometric accuracy across platforms. The mean dosimetric differences for alanine and film dose showed good agreement with expected values, however these differences showed a statistically significant variation when stratified by DRM. Geometric metrics were not significantly affected by DRM.

The introduction of dose-to-medium algorithms and DRMs has led to much discussion on their use and impact on treatment planning and dose verification [15], [16], [17]. For soft tissues, which are generally water equivalent, the impact of these algorithms and DRMs is unlikely to be significant [18]. The widespread adoption of SBRT has renewed interest in verifying doses to bone [1], [7], particularly for spinal treatments which are challenging to plan and deliver. Measured doses have contributions from the primary beam, scatter, out-of-field dose and field penumbra [5], which makes verifying delivered dose difficult. The measurements reported in this study are from doses delivered to an anthropomorphic phantom containing material equivalent to soft tissue, lung and bone.

Of note, dosimetry audit equipment was calibrated in D_w,w_ as per national guidance [19], complicating assessment of measurements reported in D_m,m_ or D_w,m_. A limitation that audited centres also face when clinically validating their D_m,m_ models in non-water equivalent materials. Further work is ongoing to develop dose-to-medium correction factors [20], [21]. DRM groups were imbalanced, with a higher proportion of D_w,w,_ reflecting clinical practice. This may reduce statistical power; however, it did not reflect study results: Statistically significant differences were observed which suggests the substantial impact of DRM choice. Non-parametric tests were used to mitigate the effects of unequal group sizes.

A key interpretive challenge is that DRM and dose calculation algorithms are not fully independent in routine clinical use: D_w,m_ and D_m,m_ require type C algorithms and D_w,w_ require type B, meaning that comparisons risk conflating reporting specification with algorithm/TPS differences. To better isolate the impact of reporting specification, centres were asked to recalculate the same plan on the same dataset using all available algorithms and DRMs, enabling within-plan comparisons that reduce confounding factors from phantom setup, measurement methodology, and inter-centre variation (Fig. 6). The strongest systematic pattern in the audit data corresponded with DRM category, with D_m,m_ and D_w,m_ showing opposing mean offsets relative to D_w,w_ measurements across both alanine and film. Spatial accuracy remained stable across DRMs. Taken together, these findings supported the interpretation that a substantial component of the observed dosimetric differences related to DRM rather than TPS/algorithm.

A potential source of variability in this work is the combination of onsite and postal audits. Onsite audits provide direct auditor oversight, whereas postal audits depend on local staff for setup, potentially increasing variability. This bias was mitigated by preloading the postal phantom with detectors prior to transit and imaging both phantom setups using local clinical protocols to provide an authentic end-to-end test.

Alanine measurements were, on average, higher than the calculated dose for all plans, but lower for D_w,m_ specifically. Underdosing the PTV is of more concern clinically than measuring higher than expected doses in the PTV for SBRT [22], [23], where a high D_max_ in the PTV is common. Only one audited plan measured below 3% of the expected dose with alanine (for both D_m,m_ and D_w,m_). There was a statistically significant difference in the measured dose when stratified by DRM, in agreement with reported measurements in bone for head and neck plans [24], where for D_m,m_ (Acuros) the average dose was 2.4% lower than D_w,w_ (AAA) and 4.1% lower than D_w,m_ (Acuros) when measured with thermoluminescent dosimeters. Another study of spine plans [25] showed a mean dose difference of 2.9% between D_m,m_ and D_w,m_ using Monte Carlo (iPlan).

The dose deviation across the 100% isodose in film indicated the dose variation over the whole PTV, whereas alanine measured only a small volume of the PTV. The film results mirrored the alanine measurements, with D_m,m_ (Alanine: 2.5%, Film: 2.6%) providing the highest dose differences and D_w,m_ (Alanine: −0.8%, Film: −2.5%) providing the lowest dose differences on average when compared to expected values. In both cases the choice of DRM was statistically significant.

Spinal SBRT necessitates exceptionally steep dose fall-off to ensure cord sparing [2], [3], [4]. This audit confirmed high geometric precision, reporting mean DTA and COD differences of 0.82 mm and 0.78 mm, respectively. With clinical PTV/PRV margins of 2–3 mm, these sub-millimetre results validated the accurate spatial positioning of the high-gradient region across all platforms. This geometric stability was particularly robust given the measurement resolution: film scanned at 72 dpi (0.35 mm pixel size) and an allowable dose grid resolution of ≤2 mm. These results approached the limits of the resolution due to the noise in the registration and calculation system, suggesting that minor variations reflected sampling limitations rather than mechanical delivery failure. DRM was not statistically significant for geometry, where evaluations spanned both bone and soft tissues. Furthermore, variable gamma pass rates align with the literature suggesting weak correlation with clinical dose-volume effects [26], [27]. This underscores prioritization of direct spatial metrics like DTA and COD in SBRT QA, where precise localization is paramount for sparing healthy tissue [19], [20]. The gamma analysis presented here has shown a wide range of passing values. Further fine-tuning of pass/fail criteria and the areas evaluated may improve utility. Gamma analysis using 5%/1 mm may be more sensitive to differences than 3%/2 mm.

The correlation matrix identified redundant metrics to streamline SBRT audit reporting. A very strong correlation (r ≈ 0.9) between 3%/2 mm and 5%/1 mm gamma criteria indicated they captured nearly identical information; however, the 5%/1 mm criterion may be more clinically appropriate for SBRT's high-precision requirements. Similarly, mean DTA correlated highly with gamma analysis, offering limited independent value. Alanine measurements and COD showed the weakest correlations, quantifying distinct aspects of delivery: alanine reflected absolute dose accuracy, essential for identifying DRM discrepancies, while COD captured systematic geometric shifts. Therefore, reporting a set of parameters consisting of alanine, 5%/1 mm gamma, and COD may provide the most holistic assessment of plan delivery.

The significant differences in the calculated dose values due to the choice of DRM suggest that different tolerance values could be used for the different DRMs, however this would make comparing benchmarking between centres complex. Therefore, correction factors for D_m,m_ and D_w,m_ are needed to compare these calculated values with measurements made with detectors calibrated in D_w,w_ [20], [21].

The results of this audit have demonstrated that the majority of centres are delivering spine SBRT consistently with acceptable dosimetric and geometric accuracy. However, it has also highlighted the challenges of measurement verification of dose calculations using D_m,m_ and D_w,m_ algorithms.

## CRediT authorship contribution statement

**Rushil Patel:** Writing – review & editing, Writing – original draft, Visualization, Validation, Software, Project administration, Methodology, Investigation, Formal analysis, Data curation, Conceptualization. **Mohammad Hussein:** Validation, Software, Formal analysis, Data curation. **Jonny Lee:** Validation, Project administration, Investigation, Data curation, Conceptualization. **Patricia Diez:** Writing – review & editing, Investigation. **Ileana Silvestre Patallo:** Writing – review & editing, Data curation. **Hannah Cook:** Data curation. **Alexandros Douralis:** Data curation. **Catharine H. Clark:** Writing – review & editing, Supervision, Methodology, Conceptualization.

## Declaration of competing interest

The authors declare that they have no known competing financial interests or personal relationships that could have appeared to influence the work reported in this paper. Audit development and a portion of the audits were funded by NHS England as part of national commissioning programmes.

## Appendix A Supplementary data

**Table S1.** Equipment combinations including Treatment Planning System (TPS), dose calculation algorithm, type, and dose reporting mode. **Table S2.** Summary statistics for dose reporting metrics including Alanine dose, gamma passing rates, mean film dose, distance-to-agreement (DTA), and center-of-dose (COD). **Supplementary Text A.** Centre-of-dose (COD) calculation methodology in VIGO.
