# Supplementary material for: Impact of dose reporting modes on dosimetric accuracy in a national end-to-end stereotactic spine radiotherapy audit
Source: Phys Imaging Radiat Oncol. 2026 Jul 14;40:101041. doi: 10.1016/j.phro.2026.101041 (PMC13444475; doi:10.1016/j.phro.2026.101041)
Supplement: Supplementary file 1 [file mmc1.pdf]

Supplementary material

Table S1: Equipment Combinations

TPS	Dose Calculation Algorithm	Type	Dose Reporting Mode
Eclipse (Varian Medical Systems, Palo Alto, CA, USA)	AAA	B	$D_{w,w}$
	Acuros	C	$D_{m,m}$ or $D_{w,m}$
Monaco (Elekta AB, Stockholm, Sweden)	Monte Carlo	C	$D_{m,m}$ or $D_{w,m}$
Pinnacle (Philips Healthcare, Fitchburg, WI, USA)	CCC	B	$D_{w,w}$
Precision (Accuray Inc., Sunnyvale, CA, USA)	Raytracing	A	$D_{w,w}$
	CCC	B	$D_{w,w}$
Raystation (RaySearch Laboratories, Stockholm, Sweden)	CCC	B	$D_{w,w}$
	Monte Carlo	C	$D_{m,m}$ or $D_{w,m}$

Table S2: Dose Reporting Metrics

Variable	Mean	Median	IQR (1/4 to 3/4)	Range (Min to Max)
Alanine Dose (Gy)	1.18	0.92	-0.30 to 2.35	-6.49 to 8.51
Gamma 3%/2mm (%)	89.45	90.45	84.22 to 97.84	68.69 to 100
Gamma 5%/1mm (%)	87.15	90.71	79.62 to 97.98	54.57 to 100
Mean Film Dose (Gy)	1.03	1.25	-1.04 to 3.64	-6.99 to 8.49
Mean DTA (mm)	0.81	0.80	0.54 to 1.04	0.35 to 1.70
COD (mm)	0.78	0.73	0.44 to 1.05	0.11 to 1.74

Supplementary A: Centre-of-dose calculation in VIGO

Take a matrix of dose points with x and y coordinates as in the image below:

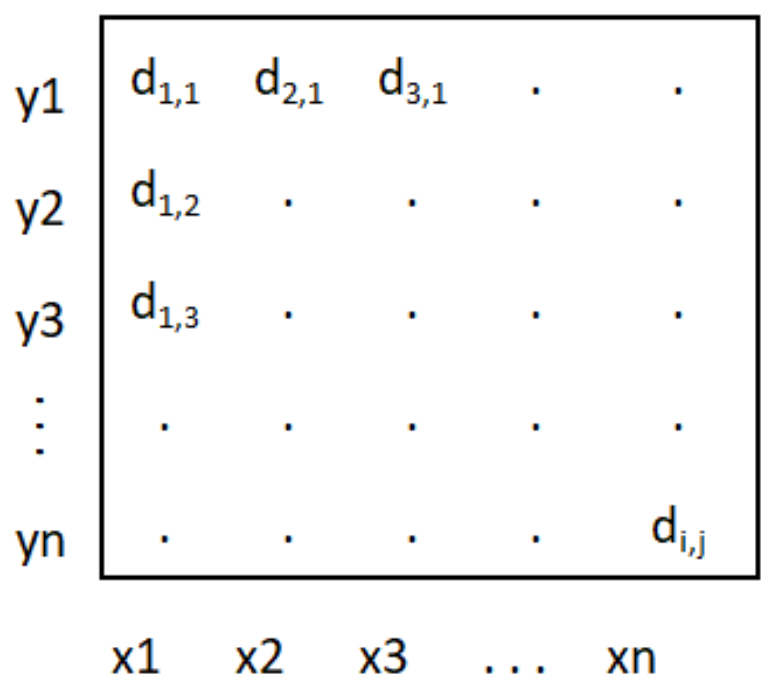

We can then work out the centre-of-dose position on the x and y axis (*COM_x* and *COM_y* respectively) using equations A.1 and A.2 respectively. This is effectively the average position of all dose points weighted by their dose value.

$$CODx = \frac{\sum_{j=1}^{n}\left(\sum_{i=1}^{n}\left(x_i \times d_{i,j}\right)\right)}{\sum_{j=1}^{n}\left(\sum_{i=1}^{n} d_{i,j}\right)} \qquad \text{(A.1)}$$

Where x_i is the position on the x-axis of each dose point at $d_{i,j}$.

$$CODy = \frac{\sum_{j=1}^{n}\left(\sum_{i=1}^{n}\left(y_j \times d_{i,j}\right)\right)}{\sum_{j=1}^{n}\left(\sum_{i=1}^{n} d_{i,j}\right)} \qquad \text{(A.2)}$$

Where y_i is the position on the y-axis of each dose point at $d_{i,j}$.

We calculate these values for each of the two datasets (e.g. for film and TPS). In the case of the SBRT analysis we include all dose points which are ≥ 100% of prescribed dose in the equations above. Then we calculate the difference using equations A.3 and A.4.

$$\Delta COD_x = COD_{x,1} - COD_{x,2} \qquad \text{(A.3)}$$

$$\Delta COD_y = COD_{y,1} - COD_{y,2} \qquad \text{(A.4)}$$

The total centre-of-dose between dataset 1 (film) and 2 (TPS) (and which is what we report) is calculated using equation A.5.

$$\Delta CODtotal = \sqrt{\Delta COD_x{}^2 + \Delta COD_y{}^2} \quad \text{(A.5)}$$

Figure A.1 shows a zoomed up isodose plot for an example SBRT plan (solid lines are Film, dashed are TPS). In this case for dose within the 100% isodose, for ΔCOD_x was -1.44mm (i.e. film is to the right of TPS) and ΔCOD_y was -1.39mm (i.e. film is anterior to the TPS centre). Hence $CODtotal$ was 2.00mm.

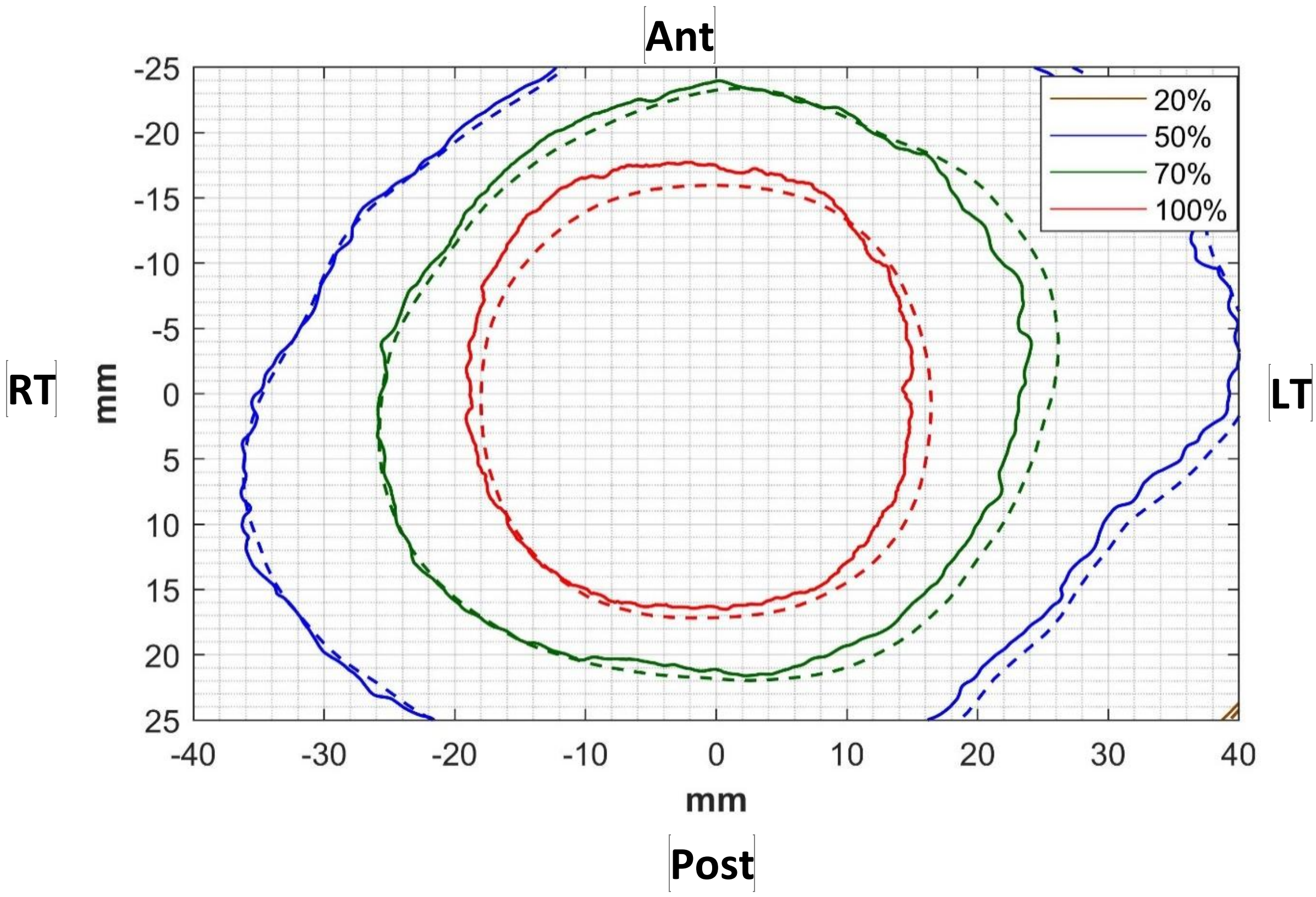

Figure A.1 Zoomed example of an isodose map with a centre-of-dose shift of 2mm.
